# Cosyntropin Attenuates Neuroinflammation in a Mouse Model of Traumatic Brain Injury

**DOI:** 10.3389/fnmol.2020.00109

**Published:** 2020-06-26

**Authors:** Lorraine Siebold, Amy C. Krueger, Jonathan A. Abdala, Johnny D. Figueroa, Brenda Bartnik-Olson, Barbara Holshouser, Christopher G. Wilson, Stephen Ashwal

**Affiliations:** ^1^Department of Basic Sciences, School of Medicine, Loma Linda University, Loma Linda, CA, United States; ^2^The Lawrence D. Longo MD Center for Perinatal Biology, Loma Linda University, Loma Linda, CA, United States; ^3^Center for Health Disparities and Molecular Medicine, School of Medicine, Loma Linda University, Loma Linda, CA, United States; ^4^Department of Radiology, Loma Linda University Medical Center, Loma Linda, CA, United States; ^5^Department of Pediatrics, Loma Linda University Medical Center, Loma Linda, CA, United States

**Keywords:** traumatic brain injury, ACTH, microglia, behavior, neuroinflammation, neutrophils, cosyntropin

## Abstract

**Aim**: Traumatic brain injury (TBI) is a leading cause of mortality/morbidity and is associated with chronic neuroinflammation. Melanocortin receptor agonists including adrenocorticotropic hormone (ACTH) ameliorate inflammation and provide a novel therapeutic approach. We examined the effect of long-acting cosyntropin (CoSyn), a synthetic ACTH analog, on the early inflammatory response and functional outcome following experimental TBI.

**Methods**: The controlled cortical impact model was used to induce TBI in mice. Mice were assigned to injury and treatment protocols resulting in four experimental groups including sham + saline, sham + CoSyn, TBI + saline, and TBI + CoSyn. Treatment was administered subcutaneously 3 h post-injury and daily injections were given for up to 7 days post-injury. The early inflammatory response was evaluated at 3 days post-injury through the evaluation of cytokine expression (IL1β and TNFα) and immune cell response. Quantification of immune cell response included cell counts of microglia/macrophages (Iba1+ cells) and neutrophils (MPO+ cells) in the cortex and hippocampus. Behavioral testing (*n* = 10–14 animals/group) included open field (OF) and novel object recognition (NOR) during the first week following injury and Morris water maze (MWM) at 10–15 days post-injury.

**Results**: Immune cell quantification showed decreased accumulation of Iba1+ cells in the perilesional cortex and CA1 region of the hippocampus for CoSyn-treated TBI animals compared to saline-treated. Reduced numbers of MPO+ cells were also found in the perilesional cortex and hippocampus in CoSyn treated TBI mice compared to their saline-treated counterparts. Furthermore, CoSyn treatment reduced IL1β expression in the cortex of TBI mice. Behavioral testing showed a treatment effect of CoSyn for NOR with CoSyn increasing the discrimination ratio in both TBI and Sham groups, indicating increased memory performance. CoSyn also decreased latency to find platform during the early training period of the MWM when comparing CoSyn to saline-treated TBI mice suggesting moderate improvements in spatial memory following CoSyn treatment.

**Conclusion**: Reduced microglia/macrophage accumulation and neutrophil infiltration in conjunction with moderate improvements in spatial learning in our CoSyn treated TBI mice suggests a beneficial anti-inflammatory effect of CoSyn following TBI.

## Introduction

Traumatic brain injury (TBI) is a major health concern in the United States resulting in a substantial number of hospitalizations and deaths (Flanagan, 2015). TBI causes subsequent morbidity and long-term effects that are influenced by age, sex, injury severity, and inflammatory status (Scherbel et al., 1999; Simon et al., 2017). Several studies have shown persistent neuroinflammation following TBI, lasting as long as 17 years (Ramlackhansingh et al., 2011; Johnson et al., 2013; Cherry et al., 2016). However, it is unclear which components of the inflammatory response are indicators of repair and which continue to drive pathology and brain vulnerability (Cherry et al., 2016). Clinically, neuroinflammation following TBI is associated with increased intracranial pressure, increased mortality, poor functional outcomes (da Silva Meirelles et al., 2017), reduced processing speed (Ramlackhansingh et al., 2011), and increased risk of epilepsy and neurodegenerative disorders (Appleton and Demellweek, 2002; Faden and Loane, 2015; Cherry et al., 2016). Together, these findings suggest that pharmaceutical agents that modulate inflammation are attractive targets to address acute and chronic symptoms of brain injury.

Under physiologically normal conditions, the brain parenchyma is isolated from the periphery by the blood-brain barrier (BBB). However, this immune privilege is severely undermined following TBI resulting in a robust immune response influenced by infiltrating peripheral immune cells (Jin et al., 2012). As the tissue-resident macrophage, microglia are key players in the inflammatory process (Xu et al., 2017). Along with microglia, peripheral monocyte, and neutrophil invasion into the brain parenchyma also contributes to the inflammatory process (Jin et al., 2012). Peripheral monocyte and neutrophil infiltration are associated with increased edema, exaggerated inflammatory responses, and poorer functional outcomes following brain injury (Kenne et al., 2012; Ma et al., 2016). Quantification of total microglia/macrophages and neutrophils show peak levels within the first week after injury with robust increases within the first few days (Jin et al., 2012; Younger et al., 2019). Due to their dynamic response to injury, tissue-resident and peripheral immune cells are essential to the early secondary injury cascade and a target for anti-inflammatory compounds.

Melanocortins are a family of peptides with diverse functions including energy homeostasis, immunomodulation, steroidogenesis, pigmentation, and synaptic plasticity (Gallo-Payet, 2016). Melanocortins (MCs) are endogenously derived from pro-opiomelanocortin precursors (Gallo-Payet, 2016). MCs include compounds such as adrenocorticotropic hormone (ACTH) and alpha-melanocyte-stimulating hormone (α-MSH), both of which are agonists for melanocortin receptors (Bertolini et al., 2009; Gallo-Payet, 2016). There are five MC receptor subtypes with tissue-specific expression, including melanocytes (MC1R), peripheral immune cells (MC1, 3, 5R), endothelial cells (MC1R), the adrenal cortex (MC2R) and the central nervous system (MC3–4R). In the central nervous system, MC receptor distribution is ubiquitous, showing expression in cortex, hippocampus, arcuate nucleus of the hypothalamus, limbic system, and thalamus (Kishi et al., 2003). Furthermore, MC receptors are expressed in neural and glial cells, contributing to their multifaceted functions (Kishi et al., 2003; Lisak et al., 2015; Chen et al., 2018). Of particular interest, MCs have been shown to have glucocorticoid (GC)-independent immune-modulatory and neuroprotective effects following brain insult (Catania et al., 2004; Catania, 2008; Montero-Melendez, 2015). Independent of MC2R activation, which results in GC production, MC1, MC3, and MC4 receptor signaling have all demonstrated neuroprotective effects (Catania et al., 2004; Schaible et al., 2013; Holloway et al., 2015; Chen et al., 2018). In these studies, MC3R and MC4R-agonist treatment reduced lesion size, inflammation, and cell death resulting in overall improvements in behavior following experimental TBI (Bitto et al., 2012; Schaible et al., 2013). In a model of cerebral ischemia-reperfusion, MC1R and MC3R signaling diminish inflammation and suppressed leukocyte recruitment following insult (Holloway et al., 2015). While the mechanisms are still unclear and are subtype and injury dependent, *in vitro* studies show that MC4R agonists reduce NFκB translocation and therefore downstream pro-inflammatory cascades, suppress pro-inflammatory microglia and protect oligodendrocytes from inflammation-related damage (Carniglia et al., 2016). MC1/3R agonists also regulate the neuroinflammatory response by reducing the expression of adhesion molecules and chemoattractants resulting in a subsequent reduction in peripheral immune cell infiltration (Scholzen et al., 2003; Holloway et al., 2015; Harazin et al., 2018). There is limited research investigating neuroprotective effects of melanocortin agonists following TBI and, to our knowledge, no research has been conducted investigating adrenocorticotrophic hormone (ACTH) or ACTH analogs as a post-TBI therapeutic (Bitto et al., 2012).

In this study, we investigated the effects of a long-acting synthetic ACTH analog (CoSyn, ACTH 1–24) on neuroinflammation and immune cell response following experimental TBI in mice and their subsequent functional outcomes. We hypothesized that CoSyn would reduce the early neuroinflammatory response and improve cognitive functioning following TBI.

## Materials and Methods

### Experimental Design

**Figure 1:**
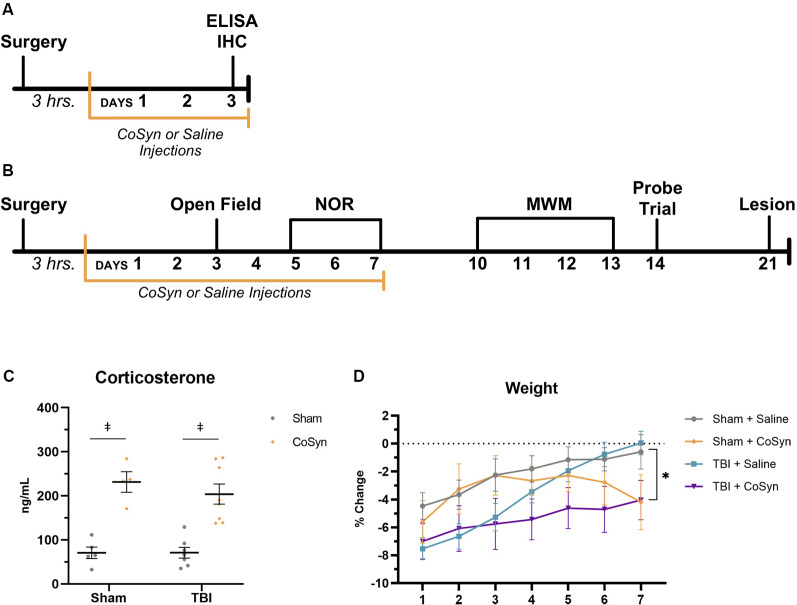
Cosyntropin (CoSyn) increases serum corticosterone levels and decreases weight gain. **(A)** Experimental design for early inflammatory response. **(B)** Experimental design for behavioral outcomes. **(C)** Serum corticosterone levels were increased at day post-injury (DPI) 3 in both sham- and traumatic brain injury (TBI)-treated animals. Sham-saline (*n* = 4), sham-CoSyn (*n* = 4), TBI-saline (*n* = 7), TBI-CoSyn (*n* = 8). **(D)** All experimental groups exhibited post-surgery weight loss. **(D)** Mice treated with CoSyn did not show post-surgical increased weight loss but did demonstrate sustained levels of weight loss at DPI 7 compared to saline-treated mice. Sham-saline (*n* = 10), sham-CoSyn (*n* = 11), TBI-saline (*n* = 14), TBI-CoSyn (*n* = 14). Significance was determined using two-way ANOVA with Tukey *post hoc* testing **(C)** and repeated-measures ANOVA **(D)**. Graphs represent means and error bars show SEM. *Main effect of treatment, *p* < 0.05, ^‡^*p* < 0.001.

Our study included two research strategies to evaluate the effect of CoSyn on the: (1) early neuroinflammatory response; and (2) behavioral outcomes following TBI (Figures 1A,B). To assess the effect of daily CoSyn administration on the early neuroinflammatory response, we quantified protein expression at day post-injury (DPI) 3 using enzyme-linked immunosorbent assays (ELISA). We also quantified microglia/macrophages and neutrophils in the ipsilateral cortex and hippocampus using immunohistochemistry at DPI 3 (Figure 1A). DPI 3 was chosen based on previous literature demonstrating robust increases in microglia/macrophages and neutrophils at this time-point (Jin et al., 2012; Younger et al., 2019). Our second research strategy included the evaluation of behavioral outcomes during and following an extended treatment protocol (Figure 1B). Taking into account both the potential for side-effects as a result of extended use (>2 weeks) and the clinical treatment protocols that suggest multiple treatment days (>5 days), we tested a 7-day protocol using a subcutaneous injection of long-acting cosyntropin (Food and Drug Administration, 2010; Antunes and Biala, 2012; Berkovich, 2013; Nasiri et al., 2017). We assessed behavior both during and following our treatment period. To assess anxiety-like behavior and memory within our treatment period, we used low anxiogenic behavioral protocols to assess anxiety-like behavior using the open field (OF) test and novel object recognition (NOR) to assess non-spatial memory (Figure 1B). Hippocampal-dependent memory was assessed through the Morris water maze (MWM, Figure 1B). Mice that underwent behavioral testing were used for DPI 21 lesion and hippocampal loss quantification.

### Animals

One-hundred and twenty-eight (128) 3-month old male C57Bl6 (Jackson Lab) mice were used for this study. Mice were housed in LLU’s Animal Care Facility on a 12-h light-dark cycle with lights turned on/off at 7:00 am/pm. Food and water were supplied *ad libitum* and food pellets were placed on the cage floor following surgery to allow access to food. Mice were randomly assigned to four experimental groups: sham + saline, sham + CoSyn, TBI + saline, or TBI + CoSyn. All procedures were approved by the Institutional Animal Care and Use Committee at Loma Linda University, Loma Linda, California. To avoid unnecessary use of experimental animals, we performed interim statistical analyses after pre-specified N values were collected.

### Controlled Cortical Impact Model

We used the controlled cortical impact model as previously described (Bajwa et al., 2016). Animals were anesthetized with isoflurane (1–3%), shaved, and the surgical area cleaned with surgical soap, 70% isopropyl alcohol, and betadine. A lidocaine injection was given before incision to expose the skull. After the skin was retracted, we made a 5.0 mm diameter craniectomy—centered between bregma and lambda and 2.5 mm lateral to the sagittal suture—to expose the underlying dura and cortex. The injury was induced with a 3.0 mm flat-tipped, metal impactor. The impactor was centered within the craniectomy site and impact occurred with a velocity of 5.3 m/s, depth of 1.5 mm, and dwell time of 100 ms. Immediately following injury, the injury site was cleaned of blood and a sterile polystyrene skull-cap was placed over the craniectomy site and sealed with VetBond (3 M, St. Paul, MN, USA). The incision was sutured and mice received an injection of saline for hydration and buprenorphine for pain prevention. Mice were placed in a heated recovery chamber and monitored for 1 h before returning to their home cage. Daily weights were taken for the first 7 days to monitor recovery. These injury parameters resulted in a severe injury composed of cortical and hippocampal loss and sustained behavioral deficits (Siebold et al., 2018). The same investigator performed all TBI and sham surgeries.

### CoSyn or Vehicle Treatment

For our treatment, we used a subcutaneous injection of long-acting cosyntropin, a synthetic analog of ACTH (amino acids 1–24) that maintains steroidogenic effects (Figure 1C). Based on previous literature, a dosage of 1.8 U/mouse/day was selected (Decker et al., 2014; Arrat et al., 2015; Cusick et al., 2015). A 3-h post-injury treatment was selected to modulate the early inflammatory response using a clinically relevant time-point (Roberts et al., 2004; Mohamadpour et al., 2019). For treatment and vehicle experimental groups, we administered CoSyn or saline treatments 3-h following cortical impact (TBI groups) or craniectomy (sham groups) with all initial treatments taking place before 19:00 on the day of surgery. Following the surgical day, mice were treated for 3 (Research Strategy 1) or 7 (Research Strategy 2) consecutive days with morning (07:00) injections. Mice were administered 50 μl of saline or CoSyn (50 μl = 1.8 units/dose) subcutaneously. CoSyn was supplied by West Therapeutic Development, LLC (Grayslake, IL, USA).

### Tissue Collection, Cytokine, and Corticosterone

For cytokine analysis, we anesthetized mice at DPI 3 and performed a cardiac puncture for blood collection. Blood was transferred to an EDTA blood collection tube for plasma isolation. Mice were then perfused with PBS and brains were quickly extracted. The hippocampus and ipsilateral cortex, containing the lesion and perilesional cortex were isolated in ice-cold PBS followed by flash-freezing in liquid nitrogen. Samples were homogenized in a protein isolation buffer with protease inhibitors (Halt Protease Inhibitor Cocktail; Sigma–Aldrich, St. Louis, MO, USA) as previously described (Fox et al., 2005). Homogenized tissue was spun down at 14 kg for 20 min at 4°C and supernatant collected. The total protein content of the supernatant was quantified using the Pierce BCA protein assay (Pierce Biotechnology, Rockford, IL, USA). IL1β and TNFα were quantified using high-sensitivity ELISA kits per manufacturer’s instructions—Mouse IL-1β/IL-1F2 Quantikine HS ELISA (Assay range 0.8–50 pg/ml, Intra-assay precision CV% <5.1) and Mouse TNFα Quantikine HS ELISA kits (Assay range 0.8–50 pg/ml, Intra-assay precision CV% <2.8, R&D Systems, Minneapolis, MN, USA). All tissue homogenates fell within the stated ranges of the high-sensitivity ELISAs. However, CoSyn-induced decreases in TNFα resulted in values below the lowest point on the standard curve (\!<0.8 pg/ml) and values were extrapolated from the standard curve. Corticosterone (CORT) levels were quantified using a Corticosterone ELISA kit with the use of steroid displacement reagent supplied with the kit (Enzo, Life Sciences, USA, sensitivity = 27 pg/ml = 20,000 pg/ml). Before quantification, serum was incubated with steroid displacement reagent to displace CORT from bound proteins and diluted to (1:100) using supplied assay buffer. All data points are averages of duplicate runs and tissue homogenates are reported as picogram (pg) of analyte per milligram (mg) of total protein.

### Immunohistochemistry

For immunostaining analysis, mice were perfused at DPI 3 with PBS followed by 4% PFA. Brains were extracted and placed in 4% PFA overnight followed by PBS washes and 30% sucrose for 48 h. Twenty-five-micrometer sections separated by 400 micrometers were cut between Bregma −2.5 and −1.0 to capture the entire lesion. Four consecutive brain sections within the lesion were used for cell count analysis. For microglia/macrophage (Iba1) and neutrophil (MPO) cell counts, staining consisted of blocking endogenous peroxide activity with quenching buffer (10% methanol, 1% hydrogen peroxide in PBS) followed by blocking with Avidin/Biotin blocking Kit (AbCam, USA) and normal serum (5% Donkey and 5% Goat serum) with 1% Triton-X in PBS. Sections were incubated overnight with a polyclonal rabbit anti-Iba1 primary antibody (1:750, Rabbit anti-Iba1, Catalog no. 019-19741, Wako, USA) or monoclonal primary anti-MPO (1:800, Rabbit anti-Iba1, Catalog no. ab208670, Abcam, USA) and followed by secondary biotinylated antibody incubation (1:200, goat anti-rabbit IgG, Vector). The Vectastain Elite ABC HRP kit and DAB peroxidase substrate kit with nickel (Vector, Burlingame, CA, USA) were used to visualize staining according to manufacturer instructions. Slides stained with anti-MPO were counter-stained with cresyl violet for reference region selection. Following staining, slides were dehydrated in ethanol and coverslipped with Permount Mounting Medium (Fisher Chemical).

### Image Acquisition, Processing, and Unbiased Stereological Analysis

Images of Iba1 and MPO stained tissue sections were acquired using the Keyence X700 (Keyence Corporation, Osaka, Japan). Images were analyzed using *ImageJ* software^1^. All cell counts were performed in a blinded fashion using an unbiased-counting methodology (Brown, 2017) developed in our laboratory and based on the unbiased stereology methods described in Mouton’s *Principles and Practices of Unbiased Stereology* (Mouton, 2002). For each manually defined reference region, *ImageJ* software was used to randomly place six boxes of equal size within the reference region. We used an unbiased dissector bounded by inclusion and exclusion lines. Cells were manually counted if their soma fell within the box or crossed the inclusion lines and were not counted if they crossed the exclusion lines (Mouton, 2002; Golub et al., 2015; Brown, 2017). For Iba1+ cell counts, each brain slice had five reference regions, including the dentate gyrus (DG), CA1, and three separate regions representing the perilesional cortex. Each cortical reference region spanned 1000 pixels (equivalent to 264 μm) in width and progressively got further from the lesion. For MPO+ cell counts, the dorsal hippocampus and perilesional cortex were defined as the two bounding reference regions incorporating these areas. Due to overt regional differences in Iba1+ cell density in the hippocampus, hippocampal Iba1+ reference regions included the dentate gyrus (DG) and CA1 regions. These regional differences were not observed for MPO+ cells, and therefore only one reference region was created for the dorsal hippocampus. For analysis, cell counts for both Iba1+ and MPO+ cells were divided by the total area of the randomly placed box and averaged across boxes and brain slices to give a final cells/area for each region.”

### Lesion Size and Hippocampal Loss

For calculation of the lesion size, every 16th section of the brain from DPI 21 mice representing a separation of approximately 400 μm and spanning the entire extent of the injured cortex were stained with cresyl violet. For cresyl violet staining, mice were perfused at DPI 21 with PBS followed by 4% PFA. Brains were extracted and placed in 4% PFA overnight followed by PBS washes and 30% sucrose for 48 h. Twenty-five-micrometer sections separated by 400 micrometers were cut between Bregma −3.5 and 1.0 to capture the entire lesion. All brain sections within Bregma −3.5 to 1.0 mm containing overt cortical lesions were used for lesion analysis. The lesion area, hippocampus, and ventricle area were calculated using the point-counting method using *ImageJ* software^1^ (Marcos et al., 2012). Regions of interest for lesion, hippocampus, and ventricles were manually drawn by technicians blinded to the experimental groups. Points acquired from the regions of interest were multiplied by the area represented by each point to get final areas for each tissue section. If the cortical loss extended into the ventricle, the total area (lesion and ventricle) was calculated and the ventricle area from the contralateral side was subtracted from the total area. Total volume was calculated using the area under the curve for each individual brain based on lesion area and separation of 400 μm per brain section.

### Behavioral Testing

All behavior testing was conducted between 16:00 and 21:00 to reduce stress-induced effects caused by 07:00 injections during the first week following injury. All mice were individually handled daily beginning 4 days before behavioral testing to acclimate mice to the investigator. All behavioral testing was conducted in cohorts that were balanced across treatment groups and were replicated with three different cohorts. Testing of behavioral set-up showed lighting levels between 31–45 lux and sound levels between 57–62 dB.

### Open Field (OF) Test

Locomotor activity and anxiety-like behavior were monitored through analysis in an open field apparatus, as previously described (Tucker et al., 2016). At DPI 3, mice were placed in a 30-cm square arena with no spatial cues (Figure 1B). Mice were gently placed in the middle of the apparatus and allowed to roam freely for 15 min. Movements were recorded and analyzed using video tracking with ANY-maze software (ANY-maze, Stoelting, Inc., Wood Dale, IL, USA).

### Novel Object Recognition (NOR)

We performed the NOR test to assess non-spatial memory performance on DPI 7 (Figure 1B; Antunes and Biala, 2012; Baratz et al., 2015). Mice were habituated for 2 days before object exposure using the same area used for the open field. No spatial cues were present. On the third day of the NOR protocol, mice were placed in the square arena along with two identical objects and were allowed to explore for 10 min and then returned to their home cage. Three hours post object exposure, mice were returned to the arena and exposed to one novel and one previous object. Objects used for previous and novel objects were balanced across experimental conditions to account for object preference. Arena and objects were thoroughly cleaned with 70% ethanol between all trials. Mice remained in the arena for 5-min before being returned to their home cage. Movements and time spent exploring were recorded, tracked, and analyzed using video tracking with ANY-maze software (ANY-maze, Stoelting, Inc., Wood Dale, IL, USA). An object discrimination ratio was calculated by dividing the total time exploring the new object by the total time exploring both objects with values greater than 0.5 indicating a preference for the novel object and therefore indicates better memory performance.

### Morris Water Maze (MWM)

As previously described, the MWM was used to assess hippocampal-dependent spatial learning and memory function (Tucker et al., 2018). The MWM apparatus was composed of a round pool filled with opaque water using non-toxic, white tempera paint with water temperatures maintained between 26–28 degrees Celsius. Black geometric shapes on a white background were placed around the pool for external spatial cues. Four days of training were conducted with five trials for each training day. On the first day of training, a flag was placed on the platform, and mice were placed on the platform for 60 s. With the flag remaining, mice were placed on the opposite side of the pool and allowed to return to the platform. The flag was removed and the mice were allowed to remain on the platform for 30 s before the next 60-s trial occurred. For all consecutive training days, mice received five 60-s trials separated by 30-s intertrial intervals. If the mice did not find the platform after the 60 s period, they were gently guided to the platform and given a latency score of 60-s for that trial. Twenty-four hours following the last training day, mice underwent a probe trial. For the probe trial, mice were placed in the pool without a platform and were allowed to swim for 60 s before removal. Following all MWM days, mice were returned to a warmed chamber to recover before being returned to their home cage. All videos were quantified and analyzed using video tracking with ANY-maze software (ANY-maze, Stoelting, Inc., Wood Dale, IL, USA).

### Statistical Analysis and Exclusion Criteria

All bar graph values in figures and text are expressed as the mean and standard error of the mean. Two-way ANOVA was used to evaluate differences in means between the four experimental groups for ELISA, cell counts, and behavior data. Repeated-measures ANOVA was used for training day analysis of the MWM and weight changes. Tukey’s multiple comparisons test was completed for *post hoc* analysis. *T*-test analysis was used for comparison of lesion size, hippocampal ratios, and MPO+ counts in the lesion. Four mice were excluded from the MWM (*n* = 2 for both TBI groups) due to the inability to swim resulting in absence of searching for an escape. Outliers were excluded from data sets using the Tukey method. Using the Tukey method, the excluded values did not exceed more than one data point per experimental group in this study. All statistical analyses were performed and graphs created with GraphPad Prism software, version 8.1.2 (GraphPad Software Inc., San Diego, CA, USA), *p* values < 0.05 were considered statistically significant.

## Results

### CoSyn Increases Serum Corticosterone and Reduces Post-injury Weight Gain

To validate drug efficacy, serum corticosterone levels were evaluated at DPI 3. CoSyn resulted in robust increases in corticosterone in serum (Figure 1C, *F*_(3,40)_ = 35.2, *p* < 0.0001) with no significant injury (*F*_(1,40)_ = 0.918, *p* = 0.344) or interaction effect (*F*_(3,40)_ = 0.313, *p* = 0.816). Elevations in corticosterone were also present in fecal matter evaluated at DPI 2, 4, and 6 (data not shown). No difference was seen in overall post-surgical percent weight-loss between treatment (*F*_(1,44)_ = 0.071, *p* = 0.791) or injury groups (Figure 1D, *F*_(1,44)_ = 3.80, *p* = 0.058). However, both CoSyn-treated groups showed sustained weight loss at DPI 7 compared to saline-treated groups (Figure 1D, *F*_(1,41)_ = 8.34, *p* = 0.0062) with no injury effect (*F*_(1,41)_ = 0.178, *p* = 0.676). TBI mice exhibited signs of distress during the first 24 h following injury including reduced movement, grooming, and nesting with no overt differences between saline and CoSyn treated mice.

### CoSyn Does Not Alter Hippocampal Loss or Cortical Lesion Volume Following TBI

**Figure 2:**
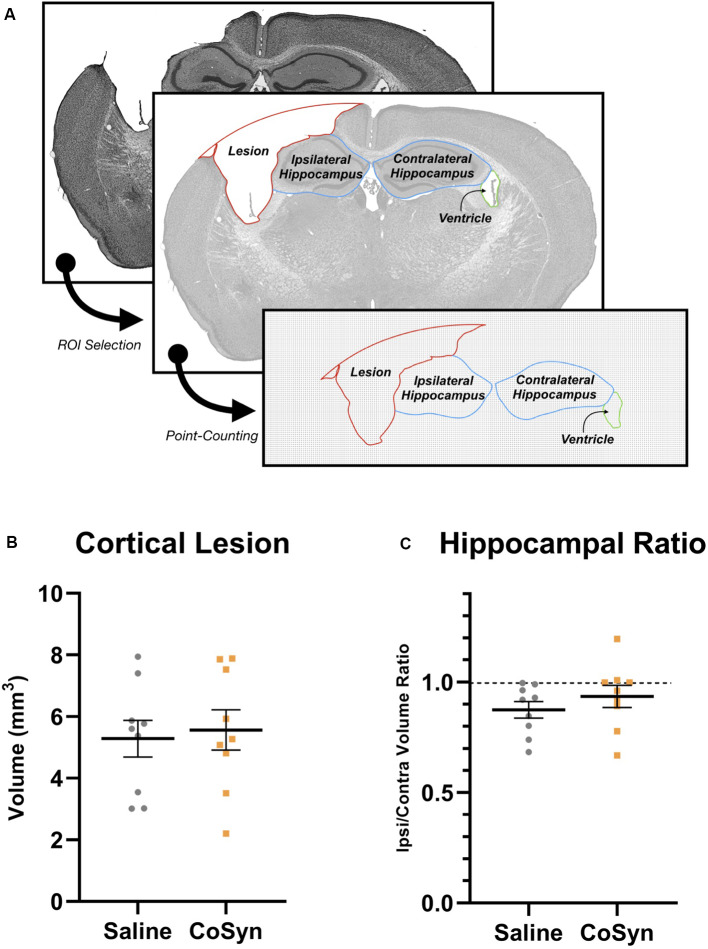
CoSyn does not alter hippocampal loss or cortical lesion size following TBI at DPI 21. **(A)** Cresyl violet stained tissue was used to quantify lesion, hippocampal, and ventricular size. **(B)** No difference was seen in cortical lesion volume between saline (*n* = 9) and CoSyn-treated (*n* = 9) mice following TBI. **(C)** CoSyn-treatment did not alter the ipsilateral/contralateral hippocampal volume ratio compared to saline-treatment. Significance was determined using an unpaired *T*-test. Graphs represent means and error bars show SEM.

To determine the effect of CoSyn on lesion size and hippocampal loss, we calculated ipsilateral cortical loss and hippocampal ratios between ipsilateral and contralateral brain regions (Figure 2A). At DPI 21, no difference was seen in cortical lesion volume between saline-treated and CoSyn-treated TBI brains (Figure 2B, *t*_(16)_ = 0.3171, *p* = 0.7553). The CoSyn-treated TBI group exhibited no difference between ipsilateral and contralateral hippocampal volume indicating no treatment-effect of CoSyn on hippocampal loss following TBI (Figure 2C, *t*_(16)_ = 0.9765, *p* = 0.3434). Our lesion volumes were consistent with previous research (Bermpohl et al., 2007).

### CoSyn Reduces Microglia/Macrophages in the Hippocampus and Cortex Following Injury

**Figure 3:**
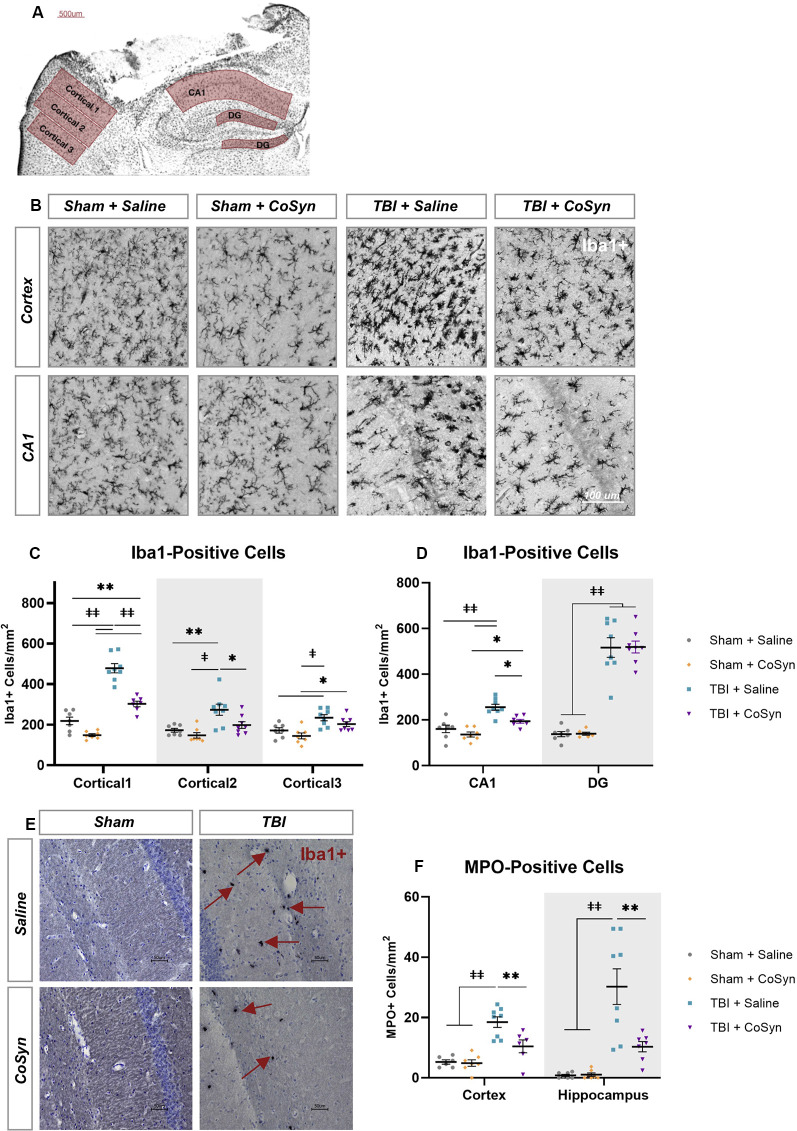
CoSyn reduced Iba1+ and MPO+ cells in the perilesional cortex and hippocampus. **(A)** Iba1+ cells were quantified at DPI 3 selected from perilesional and hippocampal regions. **(B)** Representative images of perilesional Iba1+ cells. **(C,D)** Iba1+ cells in the perilesional **(C**), as well as the CA1 and dentate gyrus (DG) regions of the hippocampus **(D)**, increased following injury. Injury-induced increases were reduced following treatment in the perilesional regions and CA1 region of the hippocampus. **(E)** Representative images of MPO+ positive cells in the hippocampus. **(F)** MPO+ cells increase after injury and are reduced with CoSyn treatment. Sham-saline (*n* = 7), sham-CoSyn (*n* = 6), TBI-saline (*n* = 9), TBI-CoSyn (*n* = 9). Significance was determined using two-way ANOVA with Tukey *post hoc* testing. Graphs represent means and error bars show SEM, *p < 0.05, **p < 0.01, ^‡^*p* < 0.001, ^‡‡^*p* < 0.0001.

Ionized calcium-binding adaptor molecule 1 (Iba1) was used to evaluate the microglial/macrophage response *via* quantification of Iba1-positive cells within the perilesional cortex and ipsilateral hippocampus (Figures 3A,B). As illustrated in Figure 3, injury induced an increase in Iba1+ cells in both the cortical regions surrounding the lesion and hippocampus (Figures 3C,D). For the perilesional cortex, we found a main effect of cortical region (Figure 3C, *F*_(2,49.37)_ = 64.29, *p* < 0.0001) and experimental group (*F*_(3,26)_ = 39.22, *p* < 0.0001) with significant interaction (*F*_(6,51)_ = 17.74, *p* < 0.0001). These differences were also seen in the hippocampus with region (Figure 3D, *F*_(1,50)_ = 81.92, *p* < 0.0001) and experimental group main effects (*F*_(3,50)_ = 71.52, *p* < 0.0001) and a significant interaction (*F*_(3,50)_ = 31.91, *p* < 0.0001). *Post hoc* analysis showed CoSyn-treatment decreased injury-induced increases of Iba1+ cells in the perilesional cortical regions (Figure 3C, *p* < 0.001) as well as the CA1 region of the hippocampus (Figure 3D, *p* = 0.0103). CoSyn did not alter the number of Iba1+ cells in the DG at DPI 3 (Figure 3D, *p* ≥ 0.999). Of interest, CoSyn-treatment in the TBI group resulted in Iba1+ cell counts equivalent to shams in cortical regions further from the lesion site while the saline-treated TBI group maintained elevated levels of Iba1+ cells in comparison to both shams in all cortical regions and the CoSyn-treated TBI group in the two regions closest to the lesion indicating a more widespread microglia/macrophage response (Figure 3C).

### CoSyn Reduces Neutrophil Infiltration in the Cortex and Hippocampus Following TBI

Following injury, MPO+ cells (Figure 3E) increased in both the perilesional cortex (Figure 3F, *F*_(1,23)_ = 36.09, *p* < 0.0001) and hippocampus (Figure 3F, *F*_(1,25)_ = 32.12, *p* < 0.0001). Main effect of treatment as well as interaction between injury and treatment were observed in both cortex (Injury effect *F*_(1,23)_ = 6.036, *p* = 0.022, Interaction *F*_(1,23)_ = 7.261, *p* = 0.0129) and hippocampus (Injury effect *F*_(1,25)_ = 8.712, *p* = 0.0068, Interaction *F*_(1,23)_ = 8.342, *p* = 0.0079). *Post hoc* analysis demonstrated CoSyn treatment reduced neutrophil infiltration in both the cortex (*p* = 0.0071) and hippocampus (*p* = 0.0018) following injury (Figure 3F). There was no difference in MPO+ cells in the lesion site when comparing saline and CoSyn treated TBI mice (data not shown).

### CoSyn Reduces IL1β in the Cortex

**Figure 4:**
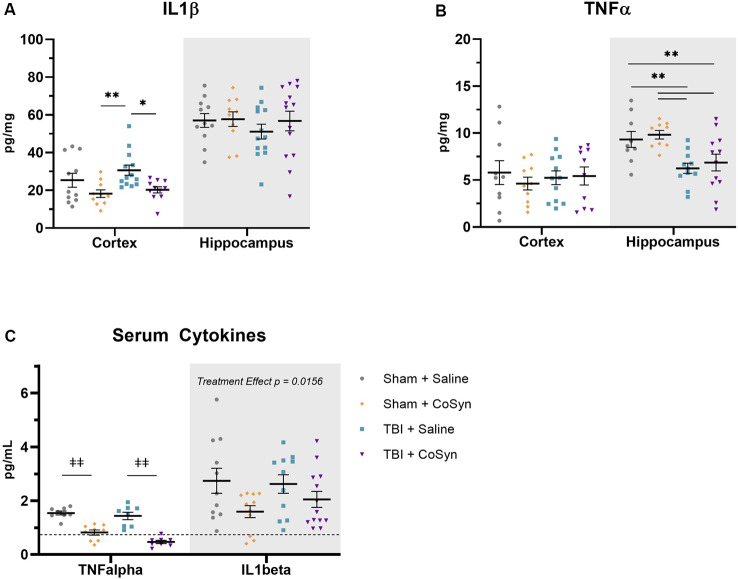
CoSyn reduces IL1β expression in cortex and serum and TNFα in serum. **(A)** Cortical IL1β levels at DPI 3 were reduced in TBI groups with CoSyn-treatment with no alteration in IL1β levels in the hippocampus between groups. **(B)** TNFα expression levels were the same between groups in the cortical tissue with lower levels exhibited in the hippocampal tissue of the TBI group compared to sham. Sham-saline (*n* = 10), sham-CoSyn (*n* = 11), TBI-saline (*n* = 13), TBI-CoSyn (*n* = 14). **(C)** TNFα and IL1β levels in serum at DPI 3, both cytokines showed decreased levels following CoSyn administration. The dashed line indicates the lowest detectable concentration for high-sensitivity ELISA. Significance was determined using two-way ANOVA with Tukey *post hoc* testing. Graphs represent means and error bars show SEM, *p < 0.05, **p < 0.01, ^‡‡^*p* < 0.001.

To test the anti-inflammatory properties of CoSyn, we evaluated expression levels of IL1β and TNFα in ipsilateral cortical and hippocampal tissue. We found that CoSyn reduced IL1β in the cortex with a main effect of treatment (Figure 4A, *F*_(1,41)_ = 10.57, *p* = 0.0023) *p* < 0.05) without altering IL1β levels in the hippocampus. *Post hoc* analysis showed reduced IL1β expression in CoSyn treated vs. Saline treated TBI mice (Figure 4A, *p* = 0.0074). We did not see differences in TNFα expression at DPI 3 between injury or treatment groups in cortical tissue (Figure 4B). Interestingly, we found that injury reduced TNFα in the hippocampus compared to sham mice with no differences seen between treatment groups (Figure 4B, *F*_(1,37)_ = 16.66, Injury effect, *p* = 0.0002). IL1β serum levels showed no injury-induced increases (Figure 4C, Injury effect *F*_(1,42)_ = 0.2434, *p* = 0.6243) but did show a treatment effect (*F*_(1,42)_ = 6.352, *p* = 0.0156) with CoSyn contributing to decreased IL1β. Similar to hippocampal tissue, serum TNFα decreased following injury (Figure 4C, *F*_(1,29)_ = 5.751, *p* = 0.0231). Unlike cortical and hippocampal tissue, CoSyn administration resulted in a robust decrease in TNFα in serum at DPI 3 (Treatment effect *F*_(1,29)_ = 78.08, *p* < 0.0001).

### TBI Increased Anxiety-Like Behavior With No Alteration in Overall Movement

**Figure 5:**
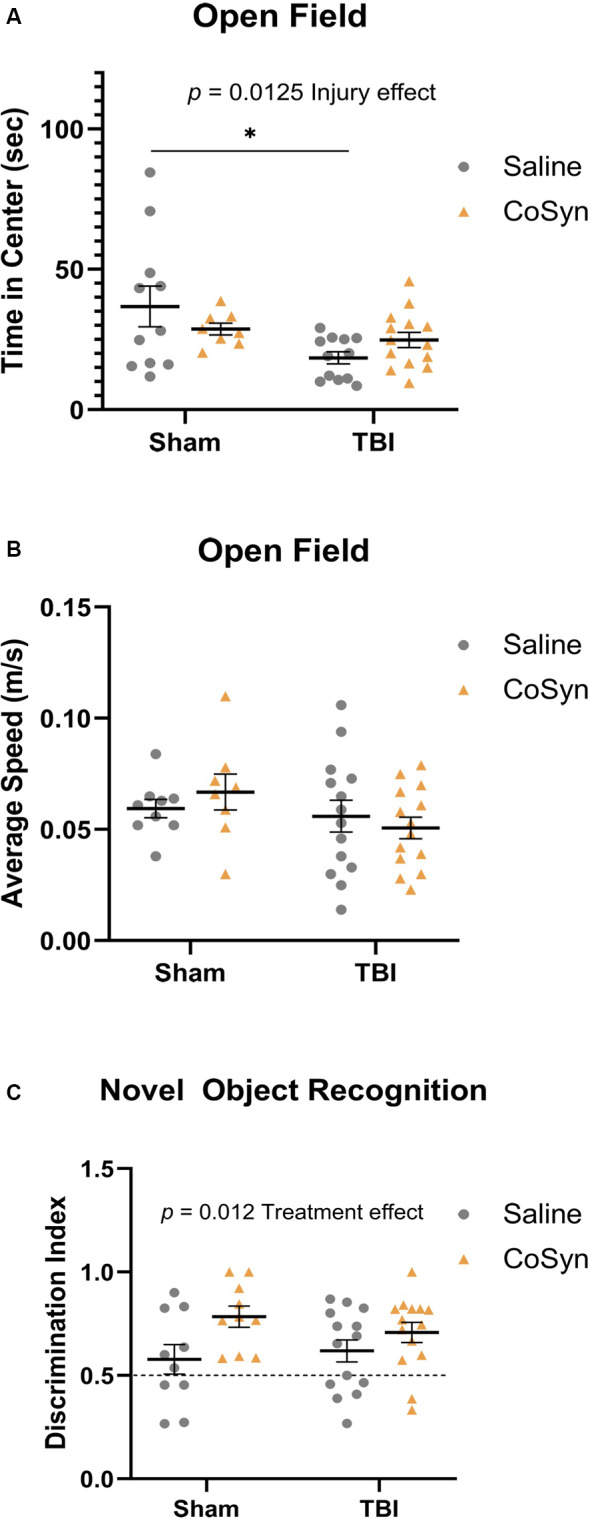
Injury-induced anxiety-like behavior and increased memory with treatment. **(A)** TBI mice exhibit reduced time in the center compare to sham animals. No effect of treatment in either group was seen. **(B)** No difference between the average speed at DPI 3 between any treatment or injury group. **(C)** CoSyn increased time-spent exploring novel objects during novel object recognition (NOR) testing as indicated by increased discrimination index. Sham-saline (*n* = 10), sham-CoSyn (*n* = 11), TBI-saline (*n* = 14), TBI-CoSyn (*n* = 14). Significance was determined using two-way ANOVA with Tukey *post hoc* testing. Graphs represent means and error bars show SEM, *p < 0.05.

Open-field testing was completed at DPI 3 to evaluate anxiety-like behavior and spontaneous motor activity. Following injury, mice exhibited reduced time spent in the center compared to shams indicating increased anxiety-like behavior (Figure 5A, *F*_(1,41)_ = 6.819, Injury effect, *p* = 0.0298). No differences were seen between saline and CoSyn treated mice (*F*_(1,41)_ = 0.04, Treatment effect, *p* = 0.842). Furthermore, no injury (*F*_(1,41)_ = 2.235, *p* = 0.143) or treatment (*F*_(1,41)_ = 0.027, *p* = 0.871) effects were seen between groups when comparing average speeds (Figure 5B).

### CoSyn Improves Non-spatial Memory

Non-spatial dependent memory was evaluated through the use of novel object recognition (NOR) testing at DPI 7. Mice did not display alterations in memory as a result of injury status (Figure 5C, *F*_(1,44)_ = 0.099, *p* = 0.755) but did show increased preference for the novel object following CoSyn-treatment (Figure 5C, *F*_(1,44)_ = 6.872, Treatment effect, *p* = 0.0120). Increased discrimination ratio suggests improved memory performance with CoSyn administration (Figure 5C).

### CoSyn Provides Modest Improvements in Early Spatial Learning Performance Following TBI

**Figure 6:**
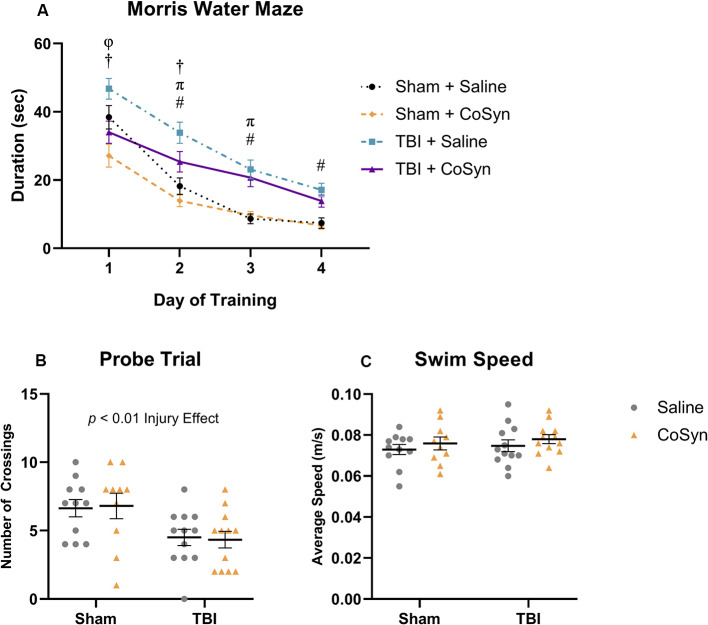
CoSyn induces modest improvements in early Morris water maze (MWM) performance post-TBI. **(A)** Mean duration to find platform across training days. All groups improved over consecutive learning days. TBI mice treated with saline spent more time finding the platform compared to TBI mice treated with CoSyn and both sham groups on training days 1 and 2. Training day comparisons with *p* < 0.05: Sham groups (φ), TBI groups (†), Saline groups (#), CoSyn groups (π). **(B)** A probe test was performed following the final training day. Both TBI groups had fewer platform crossings compared to sham groups. There was no significant difference between treated and untreated groups. **(C)** No differences were detected in swim speed between experimental groups during the probe trial. Sham-saline (*n* = 10), sham-CoSyn (*n* = 11), TBI-saline (*n* = 12), TBI-CoSyn (*n* = 12). Significance was determined using two-way ANOVA **(B,C)** and repeated-measures ANOVA **(A)** with Tukey *post hoc* testing. Graphs represent means and error bars show SEM.

To determine the effect of CoSyn on memory and learning following TBI, we conducted the Morris water maze at 10–15 days post-injury. All treatment groups learned the MWM task as indicated by a reduced latency to find the hidden platform over four consecutive days (Figure 6A). We saw both main effects for training day (*F*_(3,829)_ = 75.7, *p* < 0.0001) and experimental group (*F*_(3,829)_ = 31.19, *p* < 0.0001). *Post hoc* analysis revealed that for both treatment groups, TBI resulted in overall spatial learning deficits as demonstrated by increased latency to find the platform during the final training days compared to sham groups (Figure 6A). On the first trial day, the Sham + CoSyn group found the platform significantly faster than the Sham + Saline group (Figure 6A, *p* = 0.0204) with no difference in overall swim speed (data not shown). This pattern was replicated in the TBI groups with CoSyn-treated mice having reduced latency to find platform compared to the saline-treated TBI mice (Figure 6A, *p* = 0.0028). Sham groups showed no differences in latency to find the platform on days 2–4 of training (Figure 6A). CoSyn-treated TBI mice continued to show reduced latency time compared to saline-treated TBI mice on day two of training that did not persist through days 3–4 (Figure 6A, *p* = 0.0490). Together, these data suggest a modest improvement in spatial reference memory acquisition for TBI animals when treated with CoSyn. To assess spatial memory retention, we performed the probe test 24 h following the last training day and quantified the number of times the mice crossed the location which originally contained the platform during the training phase. We saw a reduced number of crossings in injured vs. sham groups (Figure 6B, *F*_(1,41)_ = 11.15, Injury effect, *p* = 0.0018) with no treatment effect (Figure 6B, *F*_(1,41)_ < 0.0001, *p* = 0.998). No differences were detected in average swimming speed during the probe trial (Figure 6C).

## Discussion

In this study, we investigated the effect of CoSyn administration in modulating inflammatory and behavioral outcomes following an experimental model of traumatic brain injury in mice. This is the first study to evaluate the therapeutic potential of CoSyn for the treatment of TBI. Our data showed that a 1-week regimen of CoSyn following injury resulted in improved spatial learning. At 3-days post-injury, a 3 h post-injury CoSyn treatment with daily administration attenuated accumulation of microglia/macrophages in the perilesional cortex and the CA1 region of the hippocampus and led to decreased IL1β expression in cortical tissue but did not alter cytokine expression (IL1β and TNFα) in the hippocampus. Our 1-week treatment regimen did not alter chronic cortical loss as measured by cortical lesion size 3-weeks post-injury. Improved spatial learning in conjunction with reduced microglia/macrophage accumulation and neutrophil infiltration in our CoSyn treated TBI mice suggests an immune-cell mediated therapeutic effect of CoSyn following TBI.

Current acute management following TBI includes intracranial pressure monitoring, hypertonic saline, surgical intervention, and seizure monitoring (Vella et al., 2017). While chronic inflammation is well documented in TBI patients (Ramlackhansingh et al., 2011; Johnson et al., 2013; Cherry et al., 2016), limited pharmaceutical interventions directly target the inflammatory response. Long-acting cosyntropin could be such a compound and is an attractive pharmaceutical for its multifaceted immuno-modulatory capacity. Cosyntropin also referred to as tetracosactide, is a synthetic analog composed of the first 24 amino acids of the full-length ACTH. ACTH was originally FDA approved in 1952 and, currently, two forms of ACTH have Food and Drug Administration approval for use in diagnostic testing of adrenal functioning including cosyntropin and H.P. Acthar Gel Repository Injection (Mallinckrodt Pharmaceuticals), the 39 amino acid natural form of the peptide (Gettig et al., 2009). Both the natural and synthetic variants stimulate all subtypes of the melanocortin receptors resulting in stimulation of the adrenal cortex to secrete glucocorticoids (Catania et al., 2004). The most common indications for the use of ACTH include adrenocortical testing and the treatment of multiple sclerosis (MS) and infantile spasms (Gettig et al., 2009). Due to a half-life of approximately 20 min in humans, several compounds have been developed to increase the length of activity for the natural or synthetic variants of ACTH through the use of gels or zinc suspensions (Veldhuis et al., 2001). Both the Synacthen Depot (Novartis Pharmaceuticals) and the long-acting cosyntropin supplied by West Therapeutics use a zinc suspension to extend activity. H.P. Acthar Gel (Mallinckrodt Pharmaceuticals) has FDA approval in the US and Synacthen has approval for use in several European countries (Gettig et al., 2009; Food and Drug Administration, 2010).

The most effective dosage and length of treatment for the synthetic and natural variants of ACTH remain uncertain. ACTH has been used clinically in the treatment of multiple sclerosis (MS) and infantile spasms with prolonged treatment periods extending several days to weeks (Food and Drug Administration, 2010; Berkovich, 2013; Nasiri et al., 2017). A proposed clinical algorithm for MS relapse management suggests a 5–15-day regimen of ACTH following non-response to methylprednisolone treatment (Berkovich, 2013). Extended use of ACTH in the clinic results in side effects that include Cushing’s syndrome, hypertension, ulcers, and mood disturbances (Food and Drug Administration, 2010). Taking into account both potential for side-effects as a result of extended use and the clinical treatment protocols that suggest multiple treatment days, we tested a 7-day protocol using a subcutaneous injection of long-acting cosyntropin and found reduction in early inflammatory response and improvements in cognitive functioning following TBI. Further research is needed to determine the best effective dosage and length of treatment to maximize drug efficacy and minimize side effects following TBI. Our study was limited in that we did not address dosage, route, and length of treatment regimens or chronic effects including alterations in cardiovascular health.

Furthermore, our study shows a CoSyn-induced increase in corticosterone following subcutaneous injection. Corticosterone is an agonist for glucocorticoid (GC) and mineralocorticoid receptors with differing binding affinities and dose-dependent effects on CNS function (Aharon et al., 2017; Paragliola et al., 2017). Several synthetic GC receptor agonists are widely used in the clinical setting with greater potency compared to endogenous GC agonists, including dexamethasone and methylprednisolone (Aharon et al., 2017). Unlike synthetic GC agonists, the circulating GC level induced by CoSyn is a result of MC2R activation and limited by the endogenous production capabilities of the adrenal glands. GCs have well-documented anti-inflammatory and neuroprotective effects. Some beneficial effects include decreased iNOS-mediated neurotoxicity *in vitro* (Golde et al., 2003), attenuation of LPS-induced TNFα expression (Nadeau and Rivest, 2002), and suppression of IL1β and TNFα in the hippocampus (Dinkel et al., 2003; for review see Sorrells and Sapolsky, 2007). Due to its anti-inflammatory and potential neuroprotective effects, methylprednisolone was used in a large, randomized, multi-center TBI clinical trial evaluating the effect of early administration on death and disability (Roberts et al., 2004). Results from this study indicate that high-dose methylprednisolone increases mortality following significant head injury and therefore suggests that synthetic glucocorticoids should not be used for the treatment of TBI (Roberts et al., 2004; Edwards et al., 2005). Due to increased potency of synthetic GC agonists, the use of high-dose may have contributed to increased mortality. GC actions in the CNS are dosage, timing, and regionally-specific, with both pro- and anti-inflammatory effects contributing to neuronal vulnerability and survival (Sorrells and Sapolsky, 2007). In pre-clinical studies, the supraphysiological activation of GC receptors *via* synthetic analogs has also been shown to increase mortality along with contributing to increased hippocampal cell death following TBI (Chen et al., 2009).

Additionally, GCs have been shown to impair metabolism through inhibition of glucose uptake (Sorrells and Sapolsky, 2007), reduce injury-induced BDNF (Grundy et al., 2000) and contribute to CA1 hippocampal neuronal vulnerability following TBI (McCullers et al., 2002). In the kainic acid model of CNS injury, high GC levels were also associated with increased macrophages and microglia density after 3 days of GC exposure (Dinkel et al., 2003). Conversely, glucocorticoids have also been shown to attenuate microglia/macrophage activation (Zhang et al., 2007), reduce edema and brain infarct (Campolo et al., 2013) and contribute to injury-induced increases in NGF following TBI (Grundy et al., 2001). Furthermore, as part of the HPA axis, GCs inhibit CRF and ACTH resulting in reduced expression of both hormones and chronic usage results in HPA axis suppression (Paragliola et al., 2017). As a melanocortin receptor agonist, ACTH also contributes to anti-inflammatory effects and metabolic regulation. In contrast to GCs, ACTH 1–24 increases glucose uptake in neurons through stimulation of transport protein synthesis, and MC4R-agonists increase BDNF *in vitro*, suggesting melanocortin receptor signaling may counteract deleterious effects of GC signaling (Daval et al., 1985; Saba et al., 2019). The delicate balance between CRF, ACTH, and GCs in the maintenance of homeostasis and response to stress is vital for normal functioning and is disrupted following TBI (Tapp et al., 2019). Additional research is needed to evaluate the effect of synthetic ACTH as a post-TBI therapeutic specifically addressing if its effects are dependent on its GC-inducing capacity or extend beyond this mechanism of action, potentially counteracting the side effects of supraphysiological or prolonged GC activation.

Injury-induced weight loss is typical following experimental TBI and, in healthy mice, is followed by subsequent weight gain. Our research demonstrated these trends for both sham and TBI saline-treated mice. However, CoSyn-treated mice did not exhibit compensatory weight gain over the evaluated 7-days post-injury compared to their saline-treated counterparts resulting in a treatment effect of CoSyn administration. Along with grooming, nesting, posture, and movement, weight changes are an indicator of animal health (Burkholder et al., 2012). As expected, both saline and CoSyn-treated mice displayed acute injury-induced signs of distress including hunched posture, sluggish movements, and reduced nesting behavior. Along with our current selected dosage, our lab also investigated a higher dose of CoSyn (3.4 U/mouse/day) which resulted in prolonged signs of distress including reduced grooming, sustained weight-loss, and hunched posture (data not shown). While the CoSyn-treated mice did exhibit reduced weight gain, overt changes in grooming, nesting, or posture between saline and CoSyn were not seen in the current study (1.2 U/mouse/day). Consistent with our recovery observations, the CoSyn administration did not alter the average speed in the OF test. However, lack of weight gain is concerning and additional dosage research is needed. Furthermore, the influence of ACTH and GCs on food intake, weight gain, and fat metabolism confounds the use of weight as an indicator of overall health. Prolonged exposure to increased GC levels can result in weight gain (Scerif et al., 2013; Do et al., 2019). However, the GC-induced weight gain may require a dosage regimen extending further than 7-days, as used in our study, as previous work has shown that 1 week of increased GC levels did not result in increased body weight (Do et al., 2019). Furthermore, several studies have demonstrated that central administration of ACTH results in reduced food intake (Al-Barazanji et al., 2001; Schulz et al., 2010; Shipp et al., 2015) and weight loss compared to the vehicle when given *ad libitum* access to food (Al-Barazanji et al., 2001). Reduced weight gain following post-trauma weight loss could be indicative of poor outcome, and dosage studies taking into account food consumption and metabolic rates would be beneficial.

TBI results in a complex and dynamic cytokine profile. Interleukin-1-beta (IL1β) is an early inflammatory cytokine that is restored to sham-levels within 1 week following injury and is involved in the secondary injury cascade (Dalgard et al., 2012; Lagraoui et al., 2012; Taib et al., 2017). IL1β neutralization studies demonstrated the causal role of increased IL1β levels in driving neuronal loss (Ozen et al., 2020), circulating macrophage recruitment (Basu et al., 2002), injury-induced oligodendrocyte damage (Flygt et al., 2018), and microglial activation and proliferation following TBI (Ozen et al., 2020). Collectively, these data suggest reduced IL1β is beneficial following experimental TBI. Our data did not show any alterations in IL1β expression in the hippocampus 3-days post-injury but did show a significant reduction in IL1β expression following CoSyn treatment in cortical tissue and a treatment effect of CoSyn in serum. We also did not see any IL1β expression differences between sham and TBI-mice in the hippocampus although we saw differences in Iba1+ cell counts between TBI and Sham mice in the CA1 region at this time-point. Sham surgeries result in an acute inflammatory response that diminishes rapidly compared to brain-injured mice (Lagraoui et al., 2012). We may be seeing peak levels of IL1β expression at DPI 3 without time-dependent alterations in inflammatory resolution. Reduction in IL1β expression in cortical tissue at DPI 3 is consistent with previous research indicating melanocortin agonists reduce IL1β expression and NF-kB signaling following CNS injury or an inflammatory insult (Ichiyama et al., 1999; Bitto et al., 2012; Schaible et al., 2013). No treatment or injury effect was observed with TNFα in cortical tissue. However, it is possible that we missed the window for TNFα expression increases as it has been shown to peak at 12 h post-injury and return to baseline by 18 h following injury, with no differences between sham and TBI at DPI 1 and 3 in injured cortical or hippocampal tissue (Baratz et al., 2015; Dong et al., 2016; Robinson et al., 2017). In contrast, TNFα has been reported to be increased in patients’ cerebrospinal fluid following moderate and severe TBI for 1 week, post-injury (Feng et al., 2018). Interestingly, we found a main effect of injury in both hippocampal tissue and serum, showing decreased levels of TNFα in the injured compared to the sham group. During pro-inflammatory insults, TNFα modulates hippocampal circuitry and impairs learning (Habbas et al., 2015). However, genetic inhibition of TNFα alone does not alter lesion volume or functional outcome following TBI (Bermpohl et al., 2007). Dual inhibition of TNFα and Fas are required to confer neuroprotection and improved spatial memory performance after TBI (Bermpohl et al., 2007). Thus, reduced TNFα could be a beneficial endogenous and immunosuppressive response to TBI (Mazzeo et al., 2006; Hazeldine et al., 2015).

A limitation of our study is that we only evaluated cytokine expression at one time-point. Due to the complex and time-dependent nature of cytokine expression following TBI, additional research is needed to determine the effect of CoSyn on the inflammatory resolution of cytokine expression in cortical and hippocampal tissue including earlier and later cytokine expression time-points and longitudinal changes in the cytokine storm.

Microglia activation has both beneficial and deleterious outcomes resulting from their dynamic response to injury. As demonstrated by microglia-depletion studies, microglia are neuroprotective resulting in decreased inflammation following brain injury (Jin et al., 2017). However, microglia can also exaggerate the inflammatory response through TLR4 activation and NFκB signaling (Ahmad et al., 2013; Yao et al., 2017). Sustained microglial activation also has been observed in patients who have experienced single TBIs and repeated-hit injuries that are associated with reduced cognitive functioning (Ramlackhansingh et al., 2011; Johnson et al., 2013; Cherry et al., 2016). Our observations indicate that CoSyn treatment following TBI results in reduced microglia/macrophage activation and recruitment in the perilesional cortex and CA1 region of the hippocampus. In the cortex, the microglia/macrophage response was limited in distribution indicating a more contained inflammatory response. As a result of IL1β involvement in microglia activation and proliferation as well as monocyte recruitment, our observed decreased microglia/macrophage density in the perilesional cortex and CA1 may be a result of CoSyn-mediated reduction in IL1β expression (Basu et al., 2002; Ozen et al., 2020). As the tissue-resident macrophage, microglia are key players in the inflammatory process following brain injury resulting in regenerative, phagocytic, and pro-/anti-inflammatory phenotypes (Xu et al., 2017). Microglia not only phagocytose dying neurons but also play an important role in synaptic plasticity, a process necessary for healthy cognition (Blank and Prinz, 2013). Studies of microglial activation have demonstrated that melanocortin agonists suppress microglia/macrophage activation and promote a regenerative/anti-inflammatory phenotype, corresponding to increased Arg1 and decreased IL4Rα and TLR4 gene expression as well as prevention of HMGB1 translation from the nucleus to the cytoplasm (Carniglia et al., 2016; Chen et al., 2018). Along with the suppression of microglia/macrophage proliferation and recruitment, an enhancement of regenerative microglia phenotypes may underlie the neuroprotective effects of CoSyn. Along with alterations in microglia/macrophage density, our data also demonstrate reduced neutrophil infiltration following CoSyn treatment during the early neuroinflammatory response. Neutrophils infiltrate the brain following injury contributing to the TBI pathology (Carlos et al., 1997; Kenne et al., 2012). Neutrophil infiltration exaggerates tissue loss and increases edema providing evidence for reduced neutrophil infiltration as beneficial following TBI (Kenne et al., 2012).

Learning and memory deficits are one of the most frequently reported symptoms in TBI patients—along with fatigue and headaches—and are among the most enduring and disruptive consequences (Paniak et al., 2002). We have observed injury-induced spatial memory impairments with moderate CoSyn-mediated improvement of spatial learning as demonstrated by reduced latency time to the hidden platform in the MWM. Normal learning and memory require a delicate interplay of glia and neurons to form proper circuitry and dynamic synaptic connections (Sajja et al., 2016). Following injury, hippocampal circuitry and synaptic connections are altered resulting in spatial learning deficits (Merlo et al., 2014; Logue et al., 2016). Within the hippocampus, MC4Rs have been implicated in modulating synaptic plasticity, inflammation, and cognition and may explain the effect of CoSyn on injury-induced cognitive deficits (Shen et al., 2013; Yang et al., 2016; Machado et al., 2018). Melanocortin receptor 4 is found in the hippocampus, located in CA1–3 and the DG, a brain region vulnerable following TBI and involved in spatial memory (Shen et al., 2016; Joksimovic et al., 2019). Following ischemia, an MC4R-specific agonist improved injury-induced deficits in spatial learning (Spaccapelo et al., 2011). A necessary mechanism of spatial learning is synaptic plasticity and dendritic stability in the hippocampus, which is compromised following TBI (Gao et al., 2011). In an Alzheimer’s disease model, α-MSH resulted in increased synaptic plasticity as well as rescued synaptic plasticity deficits (Shen et al., 2016). Not only do MC4R-specific agonists improve memory but ACTH-treatment in epileptic KCNA1-null mice demonstrated protection against learning and memory deficits induced by epilepsy (Scantlebury et al., 2017). ACTH administration also has been shown to reduce corticotropin-releasing factor (CRF) in the hippocampus *via* MC4R-signaling (Brunson et al., 2001; Shen et al., 2013). On the dendritic spines of the CA1 pyramidal cells, binding of CRF to CRF1 receptors leads to loss of dendritic spines which could impair synaptic plasticity and subsequent spatial learning (Shen et al., 2013). Additional research is needed to determine if alterations in synaptic plasticity are contributing to the alterations in memory performance following CoSyn-treatment in our TBI mice.

A majority of research on the benefits of melanocortins following neuroinflammatory insult and acquired brain injuries investigates MC4R-signaling *via* synthetic variants of α-MSH. However, unlike α-MSH, ACTH is the only endogenous MC that activates MC2R in the adrenal cortex, stimulating corticosteroid (CS) production (Catania et al., 2004). ACTH is superior to CS in the treatment of MS and infantile spasms with hypsarrhythmia suggesting that stimulation of endogenously produced CS does not fully explain the immune-modulatory effect of ACTH (Ross et al., 2013). CS use for the treatment of TBI has been evaluated in clinical studies with conflicting outcomes (Roberts et al., 2004; Simon et al., 2017; Vella et al., 2017). Dual activation of CS-dependent and independent responses through a synthetic ACTH analog may result in a more controlled and thorough resolution of the complex inflammatory response following TBI and may improve on current clinical trials only evaluating CS use. In this study, we have not addressed the potential role of CS production on our observed outcomes, and additional research is needed to identify the specific mechanisms of cosyntropin following TBI. Due to their varied expression in tissue and cell type, melanocortins have diverse functions documented in several reviews (Spaccapelo et al., 2011; Montero-Melendez et al., 2015; Gallo-Payet, 2016). Of relevance to acquired brain injury, these functions include modulation of energy homeostasis (Krashes et al., 2016), steroidogenesis (Goverde and Smals, 1984; Chen et al., 2007), CNS immune modulation (Huang and Tatro, 2002; Montero-Melendez et al., 2011; Bitto et al., 2012), neurogenesis and neuronal survival (Giuliani et al., 2011; Spaccapelo et al., 2011; Lisak et al., 2015), synaptic plasticity (Shen et al., 2013, 2016) and peripheral immune cell trafficking (Getting et al., 2002; Chen et al., 2018). While not within the scope of this study, the specific melanocortin receptors mediating neuroinflammatory modulation and neuroprotection following TBI are ripe for future investigation. Our study continues to support the increasing evidence for the use of melanocortin receptor agonists in the treatment of acquired brain injuries. Furthermore, melanocortin receptor agonists may have the therapeutic potential not only in the treatment of TBI but may extend to other CNS disorders including Alzheimer’s disease and chronic traumatic encephalopathy.

## Data Availability Statement

The datasets generated for this study are available on request to the corresponding author.

## Ethics Statement

The animal study was reviewed and approved by Institutional Animal Care and Use Committee at Loma Linda University, Loma Linda, CA, USA.

## Author Contributions

LS, JF, BB-O, SA, BH, and CW designed the study. LS and AK performed surgeries and data collection. LS, AK, and JA performed data analysis. LS conducted all statistical analyses and wrote the article. LS, JF, BB-O, SA, BH, and CW discussed results and revised the manuscript.

## Conflict of Interest

The authors declare that the research was conducted in the absence of any commercial or financial relationships that could be construed as a potential conflict of interest.

## Footnotes

^1^https://imagej.net/Welcome

## References

[B1] Aharon M. A. Prittie J. E. Buriko K. (2017). A review of associated controversies surrounding glucocorticoid use in veterinary emergency and critical care: glucocorticoid use in emergency and critical care. J. Vet. Emerg. Crit. Care 27, 267–277. 10.1111/vec.12603 28449321

[B2] Ahmad A. Crupi R. Campolo M. Genovese T. Esposito E. Cuzzocrea S. (2013). Absence of TLR4 reduces neurovascular unit and secondary inflammatory process after traumatic brain injury in mice. PLoS One 8:e57208. 10.1371/journal.pone.0057208 23555560 PMC3610903

[B3] Al-Barazanji K. A. Miller J. E. Rice S. Q. J. Arch J. R. S. Chambers J. K. (2001). C-terminal fragments of ACTH stimulate feeding in fasted rats. Horm. Metab. Res. 33, 480–485. 10.1055/s-2001-16941 11544562

[B4] Antunes M. Biala G. (2012). The novel object recognition memory: neurobiology, test procedure, and its modifications. Cogn. Process. 13, 93–110. 10.1007/s10339-011-0430-z 22160349 PMC3332351

[B5] Appleton R. E. Demellweek C. (2002). Post-traumatic epilepsy in children requiring inpatient rehabilitation following head injury. J. Neurol. Neurosurg. Psychiatry 72, 669–672. 10.1136/jnnp.72.5.669 11971063 PMC1737888

[B6] Arrat H. Lukas T. J. Siddique T. (2015). ACTH (acthar gel) reduces toxic SOD1 protein linked to amyotrophic lateral sclerosis in transgenic mice: a novel observation. PLoS One 10:e0125638. 10.1371/journal.pone.0125638 25955410 PMC4425507

[B7] Bajwa N. M. Halavi S. Hamer M. Semple B. D. Noble-Haeusslein L. J. Baghchechi M.. (2016). Mild concussion, but not moderate traumatic brain injury, is associated with long-term depression-like phenotype in mice. PLoS One 11:e0146886. 10.1371/journal.pone.0146886 26796696 PMC4721654

[B8] Baratz R. Tweedie D. Wang J.-Y. Rubovitch V. Luo W. Hoffer B. J.. (2015). Transiently lowering tumor necrosis factor-α synthesis ameliorates neuronal cell loss and cognitive impairments induced by minimal traumatic brain injury in mice. J. Neuroinflammation 12:45. 10.1186/s12974-015-0237-4 25879458 PMC4352276

[B9] Basu A. Krady J. K. O’Malley M. Styren S. D. DeKosky S. T. Levison S. W. (2002). The type 1 interleukin-1 receptor is essential for the efficient activation of microglia and the induction of multiple proinflammatory mediators in response to brain injury. J. Neurosci. 22, 6071–6082. 10.1523/jneurosci.22-14-06071.2002 12122068 PMC6757935

[B10] Berkovich R. (2013). Treatment of acute relapses in multiple sclerosis. Neurotherapeutics 10, 97–105. 10.1007/s13311-012-0160-7 23229226 PMC3557364

[B11] Bermpohl D. You Z. Lo E. H. Kim H.-H. Whalen M. J. (2007). TNF alpha and fas mediate tissue damage and functional outcome after traumatic brain injury in mice. J. Cereb. Blood Flow Metab. 27, 1806–1818. 10.1038/sj.jcbfm.9600487 17406655

[B12] Bertolini A. Tacchi R. Vergoni A. (2009). Brain effects of melanocortins. Pharmacol. Res. 59, 13–47. 10.1016/j.phrs.2008.10.005 18996199

[B13] Bitto A. Polito F. Irrera N. Calò M. Spaccapelo L. Marini H. R.. (2012). Protective effects of melanocortins on short-term changes in a rat model of traumatic brain injury*. Crit. Care Med. 40, 945–951. 10.1097/ccm.0b013e318236efde 22036855

[B14] Blank T. Prinz M. (2013). Microglia as modulators of cognition and neuropsychiatric disorders. Glia 61, 62–70. 10.1002/glia.22372 22740320

[B15] Brown D. L. (2017). Bias in image analysis and its solution: unbiased stereology. J. Toxicol. Pathol. 30, 183–191. 10.1293/tox.2017-0013 28798525 PMC5545670

[B16] Brunson K. L. Khan N. Eghbal-Ahmadi M. Baram T. Z. (2001). Corticotropin (ACTH) acts directly on amygdala neurons to down-regulate corticotropin-releasing hormone gene expression. Ann. Neurol. 49, 304–312. 10.1002/ana.66 11261504 PMC2849730

[B17] Burkholder T. Foltz C. Karlsson E. Linton C. G. Smith J. M. (2012). Health evaluation of experimental laboratory mice. Curr. Protoc. Mouse Biol. 2, 145–165. 10.1002/9780470942390.mo110217 22822473 PMC3399545

[B18] Campolo M. Ahmad A. Crupi R. Impellizzeri D. Morabito R. Esposito E.. (2013). Combination therapy with melatonin and dexamethasone in a mouse model of traumatic brain injury. J. Endocrinol. 217, 291–301. 10.1530/joe-13-0022 23532863

[B19] Carlos T. M. Clark R. S. B. Franicola-Higgins D. Schiding J. K. Kochanek P. M. (1997). Expression of endothelial adhesion molecules and recruitment of neutrophils after traumatic brain injury in rats. J. Leukoc. Biol. 61, 279–285. 10.1002/jlb.61.3.279 9060450

[B20] Carniglia L. Ramírez D. Durand D. Saba J. Caruso C. Lasaga M. (2016). (Nle4, D-Phe7)-α-MSh inhibits toll-like receptor (TLR)2- and TLR4-induced microglial activation and promotes a M2-Like Phenotype. PLoS One 11:e0158564. 10.1371/journal.pone.0158564 27359332 PMC4928783

[B22] Catania A. (2008). Neuroprotective actions of melanocortins: a therapeutic opportunity. Trends Neurosci. 31, 353–360. 10.1016/j.tins.2008.04.002 18550183

[B21] Catania A. Gatti S. Colombo G. Lipton J. M. (2004). Targeting melanocortin receptors as a novel strategy to control inflammation. Pharmacol. Rev. 56, 1–29. 10.1124/pr.56.1.1 15001661

[B23] Chen M. Aprahamian C. J. Kesterson R. A. Harmon C. M. Yang Y. (2007). Molecular identification of the human melanocortin-2 receptor responsible for ligand binding and signaling. Biochemistry 46, 11389–11397. 10.1021/bi700125e 17877367 PMC3216636

[B24] Chen S. Zhao L. Sherchan P. Ding Y. Yu J. Nowrangi D.. (2018). Activation of Melanocortin Receptor 4 with RO27–3225 Attenuates Neuroinflammation through AMPK/JNK/P38 MAPK Pathway after Intracerebral Hemorrhage in Mice. J. Neuroinflammation 15:106. 10.1186/s12974-018-1140-6 29642894 PMC5896146

[B25] Chen X. Zhang K.-L. Yang S.-Y. Dong J.-F. Zhang J.-N. (2009). Glucocorticoids aggravate retrograde memory deficiency associated with traumatic brain injury in rats. J. Neurotrauma 26, 253–260. 10.1089/neu.2007.0504 19236166

[B26] Cherry J. D. Tripodis Y. Alvarez V. E. Huber B. Kiernan P. T. Daneshvar D. H.. (2016). Microglial neuroinflammation contributes to tau accumulation in chronic traumatic encephalopathy. Acta Neuropathol. Commun. 4:112. 10.1186/s40478-016-0382-8 27793189 PMC5084333

[B27] Cusick M. F. Libbey J. E. Oh L. Jordan S. Fujinami R. S. (2015). Acthar gel treatment suppresses acute exacerbations in a murine model of relapsing-remitting multiple sclerosis. Autoimmunity 48, 222–230. 10.3109/08916934.2014.984836 25410153 PMC4439322

[B28] Dalgard C. L. Cole J. T. Kean W. S. Lucky J. J. Sukumar G. McMullen D. C.. (2012). The cytokine temporal profile in rat cortex after controlled cortical impact. Front. Mol. Neurosci. 5:6. 10.3389/fnmol.2012.00006 22291617 PMC3265961

[B65] da Silva Meirelles L. Simon D. Regner A. (2017). Neurotrauma: the crosstalk between neurotrophins and inflammation in the acutely injured brain. Int. J. Mol. Sci. 18:1082. 10.3390/ijms18051082 28524074 PMC5454991

[B29] Daval J.-L. Anglard P. Gerard M.-J. Vincendon G. Louis J.-C. (1985). Regulation of deoxyglucose uptake by adrenocorticotropic hormone in cultured neurons. J. Cell. Physiol. 124, 75–80. 10.1002/jcp.1041240113 2995412

[B30] Decker D. Grant C. Oh L. Becker P. M. Young D. Jordan S. (2014). Immunomodulatory effects of H.P. acthar gel on B cell development in the NZB/W F1 mouse model of systemic lupus erythematosus. Lupus 23, 802–812. 10.1177/0961203314531840 24759631

[B31] Dinkel K. MacPherson A. Sapolsky R. M. (2003). Novel glucocorticoid effects on acute inflammation in the CNS: glucocorticoids and CNS inflammation. J. Neurochem. 84, 705–716. 10.1046/j.1471-4159.2003.01604.x 12562515

[B32] Do T. T. H. Marie G. Héloïse D. Guillaume D. Marthe M. Bruno F.. (2019). Glucocorticoid-induced insulin resistance is related to macrophage visceral adipose tissue infiltration. J. Steroid Biochem. Mol. Biol. 185, 150–162. 10.1016/j.jsbmb.2018.08.010 30145227

[B33] Dong T. Zhi L. Bhayana B. Wu M. X. (2016). Cortisol-induced immune suppression by a blockade of lymphocyte egress in traumatic brain injury. J. Neuroinflammation 13:197. 10.1186/s12974-016-0663-y 27561600 PMC5000452

[B34] Edwards P. Arango M. Balica L. Cottingham R. El-Sayed H. Farrell B.. (2005). Final results of MRC CRASH, a randomised placebo-controlled trial of intravenous corticosteroid in adults with head injury—outcomes at 6 months. Lancet 365, 1957–1959. 10.1016/s0140-6736(05)66552-x 15936423

[B35] Faden A. I. Loane D. J. (2015). Chronic neurodegeneration after traumatic brain injury: Alzheimer disease, chronic traumatic encephalopathy, or persistent neuroinflammation? Neurotherapeutics 12, 143–150. 10.1007/s13311-014-0319-5 25421001 PMC4322076

[B36] Feng G. Feng J. Zhang S. Tong Y. Zhang Q. Yang X.. (2018). Altered levels of α-melanocyte stimulating hormone in cerebrospinal fluid and plasma of patients with traumatic brain injury. Brain Res. 1696, 22–30. 10.1016/j.brainres.2018.05.044 29859146

[B37] Flanagan S. R. (2015). Invited commentary on “centers for disease control and prevention report to congress: traumatic brain injury in the united states: epidemiology and rehabilitation”. Arch. Phys. Med. Rehabil. 96, 1753–1755. 10.1016/j.apmr.2015.07.001 26184889

[B38] Flygt J. Ruscher K. Norberg A. Mir A. Gram H. Clausen F.. (2018). Neutralization of interleukin-1β following diffuse traumatic brain injury in the mouse attenuates the loss of mature oligodendrocytes. J. Neurotrauma 35, 2837–2849. 10.1089/neu.2018.5660 29690837 PMC6247990

[B39] Food and Drug Administration (2010). H.P. Acthar Gel (Repository Corticotropin) Injection. Available online at: https://www.accessdata.fda.gov/drugsatfda_docs/label/2010/022432s000lbl.pdf. Accessed February 3, 2020.

[B40] Fox C. Dingman A. Derugin N. Wendland M. F. Manabat C. Ji S.. (2005). Minocycline confers early but transient protection in the immature brain following focal cerebral ischemia—reperfusion. J. Cereb. Blood Flow Metab. 25, 1138–1149. 10.1038/sj.jcbfm.9600121 15874975 PMC2262097

[B41] Gallo-Payet N. (2016). 60 years of pomc: adrenal and extra-adrenal functions of ACTH. J. Mol. Endocrinol. 56, T135–T156. 10.1530/jme-15-0257 26793988

[B42] Gao X. Deng P. Xu Z. C. Chen J. (2011). Moderate traumatic brain injury causes acute dendritic and synaptic degeneration in the hippocampal dentate gyrus. PLoS One 6:e24566. 10.1371/journal.pone.0024566 21931758 PMC3172233

[B43] Gettig J. Cummings J. P. Matuszewski K. (2009). H.P. acthar gel and cosyntropin review. PT 8, 250–257. 19561871 PMC2697107

[B44] Getting S. J. Christian H. C. Flower R. J. Perretti M. (2002). Activation of melanocortin type 3 receptor as a molecular mechanism for adrenocorticotropic hormone efficacy in gouty arthritis. Arthritis Rheum. 46, 2765–2775. 10.1002/art.10526 12384937

[B45] Giuliani D. Zaffe D. Ottani A. Spaccapelo L. Galantucci M. Minutoli L.. (2011). Treatment of cerebral ischemia with melanocortins acting at MC4 receptors induces marked neurogenesis and long-lasting functional recovery. Acta Neuropathol. 122, 443–453. 10.1007/s00401-011-0873-4 21927944

[B46] Golde S. Coles A. Lindquist J. A. Compston A. (2003). Decreased INOS synthesis mediates dexamethasone-induced protection of neurons from inflammatory injury *in vitro*. Eur. J. Neurosci. 18, 2527–2537. 10.1046/j.1460-9568.2003.02917.x 14622153

[B47] Golub V. M. Brewer J. Wu X. Kuruba R. Short J. Manchi M.. (2015). Neurostereology protocol for unbiased quantification of neuronal injury and neurodegeneration. Front. Aging Neurosci. 7:196. 10.3389/fnagi.2015.00196 26582988 PMC4628120

[B48] Goverde H. J. M. Smals A. G. H. (1984). The anomalous effect of some ACTH-fragments missing the amino acid sequence 1–10 on the corticosteroidogenesis in purified isolated rat adrenal cells. FEBS Lett. 173, 23–26. 10.1016/0014-5793(84)81009-1 6086397

[B50] Grundy P. L. Patel N. Harbuz M. S. Lightman S. L. Sharples P. M. (2000). Glucocorticoids modulate BDNF MRNA expression in the rat hippocampus after traumatic brain injury. Neuroreport 11, 3381–3384. 10.1097/00001756-200010200-00023 11059906

[B49] Grundy P. L. Patel N. Harbuz M. S. Lightman S. L. Sharples P. M. (2001). Glucocorticoids modulate the NGF MRNA response in the rat hippocampus after traumatic brain injury. Brain Res. 892, 386–390. 10.1016/s0006-8993(00)03258-3 11172788

[B51] Habbas S. Santello M. Becker D. Stubbe H. Zappia G. Liaudet N.. (2015). Neuroinflammatory TNFα impairs memory via astrocyte signaling. Cell 163, 1730–1741. 10.1016/j.cell.2015.11.023 26686654

[B52] Harazin A. Bocsik A. Barna L. Kincses A. Váradi J. Fenyvesi F.. (2018). Protection of cultured brain endothelial cells from cytokine-induced damage by α-melanocyte stimulating hormone. PeerJ 6:e4774. 10.7717/peerj.4774 29780671 PMC5958884

[B53] Hazeldine J. Lord J. M. Belli A. (2015). Traumatic brain injury and peripheral immune suppression: primer and prospectus. Front. Neurol. 6:235. 10.3389/fneur.2015.00235 26594196 PMC4633482

[B54] Holloway P. M. Durrenberger P. F. Trutschl M. Cvek U. Cooper D. Orr A. W.. (2015). Both MC_1_ and MC_3_ receptors provide protection from cerebral ischemia-reperfusion-induced neutrophil recruitment. Arterioscler. Thromb. Vasc. Biol. 35, 1936–1944. 10.1161/atvbaha.115.305348 26112010 PMC4552587

[B55] Huang Q. Tatro J. B. (2002). Alpha-melanocyte stimulating hormone suppresses intracerebral tumor necrosis factor-α and interleukin-1β gene expression following transient cerebral ischemia in mice. Neurosci. Lett. 334, 186–190. 10.1016/s0304-3940(02)01088-1 12453626

[B56] Ichiyama T. Sakai T. Catania A. Barsh G. S. Furukawa S. Lipton J. M. (1999). Systemically administered alpha-melanocyte-stimulating peptides inhibit NF-KB activation in experimental brain inflammation. Brain Res. 836, 31–37. 10.1016/s0006-8993(99)01584-x 10415402

[B58] Jin X. Ishii H. Bai Z. Itokazu T. Yamashita T. (2012). Temporal changes in cell marker expression and cellular infiltration in a controlled cortical impact model in adult male C57BL/6 Mice. PLoS One 7:e41892. 10.1371/journal.pone.0041892 22911864 PMC3404031

[B57] Jin W.-N. Shi S. X.-Y. Li Z. Li M. Wood K. Gonzales R. J.. (2017). Depletion of microglia exacerbates postischemic inflammation and brain injury. J. Cereb. Blood Flow Metab. 37, 2224–2236. 10.1177/0271678x17694185 28273719 PMC5444553

[B59] Johnson V. E. Stewart J. E. Begbie F. D. Trojanowski J. Q. Smith D. H. Stewart W. (2013). Inflammation and white matter degeneration persist for years after a single traumatic brain injury. Brain 136, 28–42. 10.1093/brain/aws322 23365092 PMC3562078

[B60] Joksimovic J. Selakovic D. Jovicic N. Mitrovic S. Mihailovic V. Katanic J.. (2019). Exercise attenuates anabolic steroids-induced anxiety via hippocampal NPY and MC4 receptor in rats. Front. Neurosci. 13:172. 10.3389/fnins.2019.00172 30863280 PMC6399386

[B61] Kenne E. Erlandsson A. Lindbom L. Hillered L. Clausen F. (2012). Neutrophil depletion reduces edema formation and tissue loss following traumatic brain injury in mice. J. Neuroinflammation 9:17. 10.1186/1742-2094-9-17 22269349 PMC3292978

[B62] Kishi T. Aschkenasi C. J. Lee C. E. Mountjoy K. G. Saper C. B. Elmquist J. K. (2003). Expression of melanocortin 4 receptor MRNA in the central nervous system of the rat. J. Comp. Neurol. 457, 213–235. 10.1002/cne.10454 12541307

[B63] Krashes M. J. Lowell B. B. Garfield A. S. (2016). Melanocortin-4 receptor-regulated energy homeostasis. Nat. Neurosci. 19, 206–219. 10.1038/nn.4202 26814590 PMC5244821

[B64] Lagraoui M. Latoche J. R. Cartwright N. G. Sukumar G. Dalgard C. L. Schaefer B. C. (2012). Controlled cortical impact and craniotomy induce strikingly similar profiles of inflammatory gene expression, but with distinct kinetics. Front. Neurol. 3:155. 10.3389/fneur.2012.00155 23118733 PMC3484408

[B66] Lisak R. P. Nedelkoska L. Bealmear B. Benjamins J. A. (2015). Melanocortin receptor agonist ACTH 1–39 protects rat forebrain neurons from apoptotic, excitotoxic and inflammation-related damage. Exp. Neurol. 273, 161–167. 10.1016/j.expneurol.2015.08.012 26300474

[B67] Logue O. C. Cramer N. P. Xu X. Perl D. P. Galdzicki Z. (2016). Alterations of functional properties of hippocampal networks following repetitive closed-head injury. Exp. Neurol. 277, 227–243. 10.1016/j.expneurol.2015.12.019 26730521

[B68] Ma Y. Li Y. Jiang L. Wang L. Jiang Z. Wang Y.. (2016). Macrophage depletion reduced brain injury following middle cerebral artery occlusion in mice. J. Neuroinflammation 13:38. 10.1186/s12974-016-0504-z 26873581 PMC4752808

[B69] Machado I. Schiöth H. B. Lasaga M. Scimonelli T. (2018). IL-1β reduces GluA1 phosphorylation and its surface expression during memory reconsolidation and α-melanocyte-stimulating hormone can modulate these effects. Neuropharmacology 128, 314–323. 10.1016/j.neuropharm.2017.09.041 29042315

[B70] Marcos R. Monteiro R. A. F. Rocha E. (2012). The use of design-based stereology to evaluate volumes and numbers in the liver: a review with practical guidelines: design-based stereology in hepatology. J. Anat. 220, 303–317. 10.1111/j.1469-7580.2012.01475.x 22296163 PMC3375768

[B71] Mazzeo A. T. Kunene N. K. Gilman C. B. Hamm R. J. Hafez N. Bullock M. R. (2006). Severe human traumatic brain injury, but not cyclosporin A treatment, depresses activated T lymphocytes early after injury. J. Neurotrauma 23, 962–975. 10.1089/neu.2006.23.962 16774480

[B72] McCullers D. L. Sullivan P. G. Scheff S. W. Herman J. P. (2002). Mifepristone protects CA1 hippocampal neurons following traumatic brain injury in rat. Neuroscience 109, 219–230. 10.1016/s0306-4522(01)00477-8 11801359

[B73] Merlo L. Cimino F. Angileri F. F. La Torre D. Conti A. Cardali S. M.. (2014). Alteration in synaptic junction proteins following traumatic brain injury. J. Neurotrauma 31, 1375–1385. 10.1089/neu.2014.3385 24661152

[B74] Mohamadpour M. Whitney K. Bergold P. J. (2019). The importance of therapeutic time window in the treatment of traumatic brain injury. Front. Neurosci. 13:07. 10.3389/fnins.2019.00007 30728762 PMC6351484

[B76] Montero-Melendez T. Gobbetti T. Cooray S. N. Jonassen T. E. N. Perretti M. (2015). Biased agonism as a novel strategy to harness the proresolving properties of melanocortin receptors without eliciting melanogenic effects. J. Immunol. 194, 3381–3388. 10.4049/jimmunol.1402645 25725103

[B75] Montero-Melendez T. Patel H. B. Seed M. Nielsen S. Jonassen T. E. N. Perretti M. (2011). The melanocortin agonist AP214 exerts anti-inflammatory and proresolving properties. Am. J. Pathol. 179, 259–269. 10.1016/j.ajpath.2011.03.042 21703408 PMC3123798

[B77] Montero-Melendez T. (2015). ACTH: the forgotten therapy. Semin. Immunol. 27, 216–226. 10.1016/j.smim.2015.02.003 25726511

[B79] Mouton P. R. (2002). Principles and Practices of Unbiased Stereology: An Introduction for Bioscientists. MD, USA: Johns Hopkins University Press.

[B80] Nadeau S. Rivest S. (2002). Endotoxemia prevents the cerebral inflammatory wave induced by intraparenchymal lipopolysaccharide injection: role of glucocorticoids and CD14. J. Immunol. 169, 3370–3381. 10.4049/jimmunol.169.6.3370 12218159

[B81] Nasiri J. Sarajan A. Salari M. Sedghi M. (2017). Therapeutic effects of adrenocorticotropic hormone ACTH in children with severely intractable seizure. Iran. J. Child Neurol. 11, 19–26. 28883872 PMC5582355

[B82] Ozen I. Ruscher K. Nilsson R. Flygt J. Clausen F. Marklund N. (2020). Interleukin-1 beta neutralization attenuates traumatic brain injury-induced microglia activation and neuronal changes in the globus pallidus. Int. J. Mol. Sci. 21:387. 10.3390/ijms21020387 31936248 PMC7014296

[B83] Paniak C. Reynolds S. Phillips K. Toller-Lobe G. Melnyk A. Nagy J. (2002). Patient complaints within 1 month of mild traumatic brain injury: a controlled study. Arch. Clin. Neuropsychol. 17, 319–334. 10.1016/s0887-6177(01)00115-9 14589717

[B84] Paragliola R. M. Papi G. Pontecorvi A. Corsello S. M. (2017). Treatment with synthetic glucocorticoids and the hypothalamus-pituitary-adrenal axis. Int. J. Mol. Sci. 18:2201. 10.3390/ijms18102201 29053578 PMC5666882

[B85] Ramlackhansingh A. F. Brooks D. J. Greenwood R. J. Bose S. K. Turkheimer F. E. Kinnunen K. M.. (2011). Inflammation after trauma: microglial activation and traumatic brain injury. Ann. Neurol. 70, 374–383. 10.1002/ana.22455 21710619

[B86] Roberts I. Yates D. Sandercock P. Farrell B. Wasserberg J. Lomas G.. (2004). Effect of intravenous corticosteroids on death within 14 days in 10008 adults with clinically significant head injury (MRC CRASH Trial): randomized placebo-controlled trial. Lancet 364, 1321–1328. 10.1016/S0140-6736(04)17188-2 15474134

[B87] Robinson S. Berglass J. B. Denson J. L. Berkner J. Anstine C. V. Winer J. L.. (2017). Microstructural and microglial changes after repetitive mild traumatic brain injury in mice: microstructure in repetitive mild brain injury. J. Neurosci. Res. 95, 1025–1035. 10.1002/jnr.23848 27452502 PMC5266745

[B88] Ross A. P. Ben-Zacharia A. Harris C. Smrtka J. (2013). Multiple sclerosis, relapses and the mechanism of action of adrenocorticotropic hormone. Front. Neurol. 4:21. 10.3389/fneur.2013.00021 23482896 PMC3591751

[B89] Saba J. Carniglia L. Ramírez D. Turati J. Imsen M. Durand D.. (2019). Melanocortin 4 receptor activation protects striatal neurons and glial cells from 3-nitropropionic acid toxicity. Mol. Cell. Neurosci. 94, 41–51. 10.1016/j.mcn.2018.12.002 30529228

[B90] Sajja V. S. S. S. Hlavac N. VandeVord P. J. (2016). Role of glia in memory deficits following traumatic brain injury: biomarkers of glia dysfunction. Front. Integr. Neurosci. 10:7. 10.3389/fnint.2016.00007 26973475 PMC4770450

[B91] Scantlebury M. H. Chun K.-C. Ma S.-C. Rho J. M. Kim D. Y. (2017). Adrenocorticotropic hormone protects learning and memory function in epileptic Kcna1-null mice. Neurosci. Lett. 645, 14–18. 10.1016/j.neulet.2017.02.069 28249786 PMC5774237

[B92] Scerif M. Füzesi T. Thomas J. D. Kola B. Grossman A. B. Fekete C.. (2013). CB1 receptor mediates the effects of glucocorticoids on AMPK activity in the hypothalamus. J. Endocrinol. 219, 79–88. 10.1530/joe-13-0192 23884964

[B93] Schaible E.-V. Steinsträßer A. Jahn-Eimermacher A. Luh C. Sebastiani A. Kornes F.. (2013). Single administration of tripeptide α-MSH(11–13) attenuates brain damage by reduced inflammation and apoptosis after experimental traumatic brain injury in mice. PLoS One 8:e71056. 10.1371/journal.pone.0071056 23940690 PMC3733710

[B94] Scherbel U. Raghupathi R. Nakamura M. Saatman K. E. Trojanowski J. Q. Neugebauer E.. (1999). Differential acute and chronic responses of tumor necrosis factor-deficient mice to experimental brain injury. Proc. Natl. Acad. Sci. U S A 96, 8721–8726. 10.1073/pnas.96.15.8721 10411942 PMC17583

[B95] Scholzen T. E. Sunderkötter C. Kalden D.-H. Brzoska T. Fastrich M. Fisbeck T.. (2003). α-melanocyte stimulating hormone prevents lipopolysaccharide-induced vasculitis by down-regulating endothelial cell adhesion molecule expression. Endocrinology 144, 360–370. 10.1210/en.2002-220651 12488365

[B96] Schulz C. Paulus K. Lobmann R. Dallman M. Lehnert H. (2010). Endogenous ACTH, not only α-melanocyte-stimulating hormone, reduces food intake mediated by hypothalamic mechanisms. Am. J. Physiol. Endocrinol. Metab. 298, E237–E244. 10.1152/ajpendo.00408.2009 19920221

[B97] Shen Y. Fu W.-Y. Cheng E. Y. L. Fu A. K. Y. Ip N. Y. (2013). Melanocortin-4 receptor regulates hippocampal synaptic plasticity through a protein kinase A-dependent mechanism. J. Neurosci. 33, 464–472. 10.1523/jneurosci.3282-12.2013 23303927 PMC6704894

[B98] Shen Y. Tian M. Zheng Y. Gong F. Fu A. K. Y. Ip N. Y. (2016). Stimulation of the hippocampal POMC/MC4R circuit alleviates synaptic plasticity impairment in an Alzheimer’s disease model. Cell Rep. 17, 1819–1831. 10.1016/j.celrep.2016.10.043 27829153

[B99] Shipp S. L. Yi J. Dridi S. Gilbert E. R. Cline M. A. (2015). The central anorexigenic mechanism of adrenocorticotropic hormone involves the caudal hypothalamus in chicks. Neuropeptides 53, 29–35. 10.1016/j.npep.2015.07.005 26297349

[B100] Siebold L. Obenaus A. Goyal R. (2018). Criteria to define mild, moderate and severe traumatic brain injury in the mouse controlled cortical impact model. Exp. Neurol. 310, 48–57. 10.1016/j.expneurol.2018.07.004 30017882

[B101] Simon D. W. McGeachy M. J. Bayır H. Clark R. S. B. Loane D. J. Kochanek P. M. (2017). The far-reaching scope of neuroinflammation after traumatic brain injury. Nat. Rev. Neurol. 13, 171–191. 10.1038/nrneurol.2017.13 28186177 PMC5675525

[B102] Sorrells S. F. Sapolsky R. M. (2007). An inflammatory review of glucocorticoid actions in the CNS. Brain Behav. Immun. 21, 259–272. 10.1016/j.bbi.2006.11.006 17194565 PMC1997278

[B103] Spaccapelo L. Bitto A. Galantucci M. Ottani A. Irrera N. Minutoli L.. (2011). Melanocortin MC4 receptor agonists counteract late inflammatory and apoptotic responses and improve neuronal functionality after cerebral ischemia. Eur. J. Pharmacol. 670, 479–486. 10.1016/j.ejphar.2011.09.015 21946115

[B104] Taib T. Leconte C. Van Steenwinckel J. Cho A. H. Palmier B. Torsello E.. (2017). Neuroinflammation, myelin and behavior: temporal patterns following mild traumatic brain injury in mice. PLoS One 12:e0184811. 10.1371/journal.pone.0184811 28910378 PMC5599047

[B105] Tapp Z. M. Godbout J. P. Kokiko-Cochran O. N. (2019). A tilted axis: maladaptive inflammation and HPA axis dysfunction contribute to consequences of TBI. Front. Neurol. 10:345. 10.3389/fneur.2019.00345 31068886 PMC6491704

[B1055] Tucker L. B. Amanda H. F. Joseph T. M. (2016). Performance of male and female C57BL/6J mice on motor and cognitive tasks commonly used in pre-clinical traumatic brain injury research. J. Neurotrauma 33, 880–894. 10.1089/neu.2015.3977 25951234 PMC4860656

[B10555] Tucker L. B. Alexander G. V. Joseph T. M. (2018). Applications of the morris water maze in translational traumatic brain injury research. Neuroscience & Biobehavioral Reviews 88, 187–200. 10.1016/j.neubiorev.2018.03.010 29545166

[B106] Veldhuis J. D. Iranmanesh A. Naftolowitz D. Tatham N. Cassidy F. Carroll B. J. (2001). Corticotropin secretory dynamics in humans under low glucocorticoid feedback. J. Clin. Endocrinol. Metab. 86, 5554–5563. 10.1210/jcem.86.11.8046 11701735

[B107] Vella M. A. Crandall M. L. Patel M. B. (2017). Acute management of traumatic brain injury. Surg. Clin. North Am. 97, 1015–1030. 10.1016/j.suc.2017.06.003 28958355 PMC5747306

[B108] Xu H. Wang Z. Li J. Wu H. Peng Y. Fan L.. (2017). The polarization states of microglia in TBI: a new paradigm for pharmacological intervention. Neural Plast. 2017:5405104. 10.1155/2017/5405104 28255460 PMC5309408

[B109] Yang J. Qi F. Gu H. Zou J. Yang Y. Yuan Q.. (2016). Neonatal BCG vaccination of mice improves neurogenesis and behavior in early life. Brain Res. Bull. 120, 25–33. 10.1016/j.brainresbull.2015.10.012 26536170

[B110] Yao X. Liu S. Ding W. Yue P. Jiang Q. Zhao M.. (2017). TLR4 signal ablation attenuated neurological deficits by regulating microglial M1/M2 phenotype after traumatic brain injury in mice. J. Neuroimmunol. 310, 38–45. 10.1016/j.jneuroim.2017.06.006 28778443

[B111] Younger D. Murugan M. Rama Rao K. V. Wu L.-J. Chandra N. (2019). Microglia receptors in animal models of traumatic brain injury. Mol. Neurobiol. 56, 5202–5228. 10.1007/s12035-018-1428-7 30554385

[B112] Zhang Z. Zhang Z. Artelt M. Burnet M. Schluesener H. J. (2007). Dexamethasone attenuates early expression of three molecules associated with microglia/macrophages activation following rat traumatic brain injury. Acta Neuropathol. 113, 675–682. 10.1007/s00401-007-0195-8 17265048

